# Chronic Psychological Stress Induces the Accumulation of Myeloid-Derived Suppressor Cells in Mice

**DOI:** 10.1371/journal.pone.0074497

**Published:** 2013-09-18

**Authors:** Jianfeng Jin, Xiaoqian Wang, Qingyang Wang, Xiangrui Guo, Junxia Cao, Xueying Zhang, Ting Zhu, Dalin Zhang, Wendie Wang, Jing Wang, Beifen Shen, Xu Gao, Yanchun Shi, Jiyan Zhang

**Affiliations:** 1 Department of Molecular Immunology, Institute of Basic Medical Sciences, Beijing, P. R. China; 2 Department of Biochemistry and Molecular Biology, Harbin Medical University, Harbin, P. R. China; 3 Research Center of Molecular Biology, Inner Mongolia Medical College, Hohhot, P. R. China; Rutgers - New Jersey Medical School, United States of America

## Abstract

Chronic psychological stress has been shown to adversely impact immune system functions and compromise host defenses against various infections. However, the underlying mechanisms remain elusive. Recent studies have demonstrated that myeloid-derived suppressor cells (MDSCs) play an important role in regulating immunity. It is of interest to explore whether or not chronic psychological stress plays immunosuppressive functions partially by inducing MDSCs accumulation. In this work, we report that chronic psychological stress led to the accumulation of CD11b+Gr1+ cells in the bone marrow of BALB/c mice. Repeated β-agonist infusion showed no such effect. However, β-adrenergic blockade, but not glucocorticoids blockade, partially reversed the accumulation of CD11b+Gr1+ cells under the condition of chronic psychological stress, suggesting catecholamines collaborate with other factors to induce the accumulation. Further exploration indicates that cyclooxygenase 2 (COX-2)-prostaglandin E_2_ (PGE_2_) loop might act downstream to induce the accumulation. A majority of the accumulated CD11b+Gr1+ cells were Ly6G+Ly6C^low^ immature neutrophils, which inhibited cytokine release of macrophages as well as T cell responsiveness. Moreover, the accumulated CD11b+Gr1+ cells under the condition of chronic psychological stress expressed multiple inhibitory molecules. Taken together, our data demonstrate for the first time that chronic psychological stress induces MDSCs accumulation in mice, which can contribute to immunosuppression.

## Introduction

More and more demands and stimuli continuously enhance people’s psychological stress level. A psychological stress response is short lasting and adaptive processes occur very rapidly in the stressed body. However, if individuals are repeatedly stressed, neuroendocrine dysregulation can be prolonged and might cause disease. Psychological stress, if sustained, can adversely affect critical functions such as immune surveillance [1-3], gastrointestinal integrity [4], coronary artery disease [5], and wound healing [6,7]. In addition, chronic psychological stress might eventually compromise host defenses against bacterial and viral infections [8-15]. Chronic psychological stress is associated with persistent activation of the hypothalamic-pituitary-adrenal (HPA) axis, which leads to continuously elevated levels of stress hormones such as glucocorticoid and catecholamines [11,16,17]. Despite that recent studies have shown the reduction of various effectors and/or a functional compromise of such effectors caused by increased plasma levels of endogenous glucocorticoids and catecholamines mediate the immunosuppressive effects of chronic psychological stress [2,3,13,14], the pathogenic mechanisms underlying the negative impact of chronic psychological stress on host defenses against infection remain elusive.

Recently, myeloid-derived suppressor cells (MDSCs) have gained lots of attention because they potently perturb both innate and adaptive immune responses. In the mouse, the MDSCs populations have been divided into two groups: polymorphonuclear MDSCs (PMN-MDSCs) described as CD11b+Gr1^high^Ly6G+Ly6C^low/int^ cells and mononuclear MDSCs (Mo-MDSCs) described as CD11b+Gr1^int^Ly6G-Ly6C^high^ cells [18]. MDSCs were originally described in the contexts of murine tumor models and cancer patients. Elevated levels of prostaglandin E_2_ (PGE_2_) and vascular endothelial growth factor (VEGF) in tumor microenvironment have been identified to induce the expansion of MDSCs [19-21] Over the last few years, it has become appreciated that MDSCs participate in a variety of inflammatory immune responses and accumulate in spleens of mice in various models of immunosuppression [22,23]. MDSCs might express programmed death ligand (PD-L), Fas ligand (FasL), interleukin (IL)-10, Arginase I, inducible nitric oxide synthase (iNOS), and reactive oxygen species (ROS), which have been attributed to the MDSCs-mediated suppression of immune responses [18,24]. It remains unknown whether MDSCs contribute to the immunosuppressive effects of chronic psychological stress. Since it has been reported that stressful life events are associated with altered levels of MDSCs in eight breast cancer patients [25], we hypothesize that chronic psychological stress leads to elevated levels of MDSCs. In the present study, we investigated the relationship between psychological stress and MDSCs, and found that chronic psychological stress leads to the accumulation of PMN-MDSCs in the bone marrow of BALB/c mice.

## Materials and Methods

### 1: Mice

Female BALB/c mice, 6-8 weeks, were purchased from Institute of Experimental Animals, Academy of Chinese Medical Sciences. All mice were maintained under specific pathogen-free conditions. The care, use and treatment of mice in this study was in strict agreement with guidelines in the care and use of laboratory animal manual set out by the Institute of Basic Medical Sciences. The protocol was approved by the Institute of Basic Medical Sciences. All surgery was performed under sodium pentobarbital anesthesia, and all efforts were made to minimize suffering.

### 2: Reagents

Epinephrine, isoproterenol, propranolol, mifeprostone (RU486), antalarmin, SC-236, lipopolysaccharide (LPS), and Brewer thioglycollate medium were purchased from Sigma Chemical Co. (St. Louis, MO). Fetal bovine serum (FBS) was purchased from HyClone Laboratories (Logan, UT). Recombinant human Macrophage colony-stimulating factor (M-CSF) was purchased from Cetus Corp. (Emeryville, CA). All ELISA kits and PE-, PE-Cy5-, or FITC-conjugated antibodies against CD11b, Gr1, PD-L1, PD-L2, and FasL were purchased from eBioscience (San Diego, CA). Anti-PE microbeads, CD4+ T cell isolation kit II, anti-CD25 microbeads, and MS Columns were purchased from Miltenyi Biotech (Bergisch Gladbach, Germany). Antibodies against cyclooxygenase 2 (COX-2) and β-actin were from Santa Cruz Biotechnology (Santa Cruz, CA). Image-iT^TM^ live green reactive oxygen species detection kit, carboxyfluorescein diacetate succinimidyl ester (CFSE), TRIzol, Oligo dT primers, M-MLV reverse transcriptase, and SYBR Green reagent were from Invitrogen (Carlsbad, CA). ECL chemiluminescence kit was from Amersham (Arlington Heights, IL).

### 3: Chronic psychological stress

Each mouse subjected to restraint was placed in a well-ventilated 50-ml conical tube containing approximately one hundred 0.4-cm diameter holes. They were restrained for 5 consecutive nights during their active period each day (restraint stress, from 17: 00 to 09:00). The restraint tubes were cleaned and sterilized between each restraint cycles. Since mice in the tubes did not have access to food and water during this time period, similarly food- and water-deprived, but not restrained, mice were used as control animals [2,3,7,9,11,14,15]. To explore the effects of glucocorticoids, mice were intraperitoneally (i.p) injected with 25mg/kg RU486 and 10mg/kg antalarmin, or same volume of propylene glycol/ethanol (7/3 v/v) for five days. RU486 and antalarmin were dissolved in propylene glycol/ethanol (7/3 v/v). To explore the effects of catecholamines, mice were injected i.p with 2mg/kg epinephrine, 50mg/kg isoproterenol, 10mg/kg propranolol, or same volume of 0.9% saline for five days. Epinephrine, isoproterenol, and propranolol were dissolved in 0.9% saline. To explore the effects of COX-2, mice were injected with 10mg/kg SC-236 or same volume of dimethyl sulfoxide (DMSO). SC-236 was dissolved in DMSO. Mice injected with epinephrine or isoproterenol did not undergo restraint stress. Mice injected with RU486 and antalarmin, propranolol, or SC-236 underwent restraint stress 1 hour after the injection.

### 4: Flow cytometry

Cells were stained with PE-, PE-Cy5-, or FITC-conjugated antibodies in PBS containing 0.1% sodium azide and 2% FBS for 30 min on ice in the dark. Samples were washed once in staining buffer. Then the cells were fixed with 1% (w/v) paraformaldehyde in PBS and preserved at 4 °C. Isotype antibodies were included as negative control. Flow cytometry was carried out on a Becton-Dickinson FACSCalibur machine (BD Biosciences, Franklin Lakes, NJ).

### 5: Purification of Gr1+ bone marrow cells

Single-cell suspensions of bone marrow were stained with PE-anti-Gr1, followed by incubation with anti-PE microbeads at 4°C for 15 min. All Gr1+ cells were harvested with an MS column. At least 95% of the harvested cells were CD11b+Gr1+ cells as confirmed by flow cytometry.

### 6: Induction of bone marrow-derived macrophages

Mouse bone marrow cells from female BALB/c mice 6-8 weeks of age were collected as previously described [26]. The non-adherent bone marrow cells (5 × 10^5^/1.5ml/well) were then cultured for 7 days in 6-well plates at 37 °C in a humidified atmosphere containing 5% CO_2_ in RPMI 1640 medium containing 10% (v/v) FBS, 2 mM L-glutamine, 100U/ml penicillin, 100µg/ml streptomycin, 50µM β-mercaptoethanol and 10ng/ml recombinant human M-CSF.

### 7: *In vitro* suppression assays of cytokine production from macrophages

Purified bone marrow Gr1+ cells were co-cultured with bone marrow-derived macrophages in a 96 well plate at the ratio of 4:1 in the absence or presence of LPS. 24 hours later, supernatants were harvested and subjected to ELISA. In all the wells, the number of bone marrow-derived macrophages was kept constant (1×10^5^ cells/well).

**Figure 1:**
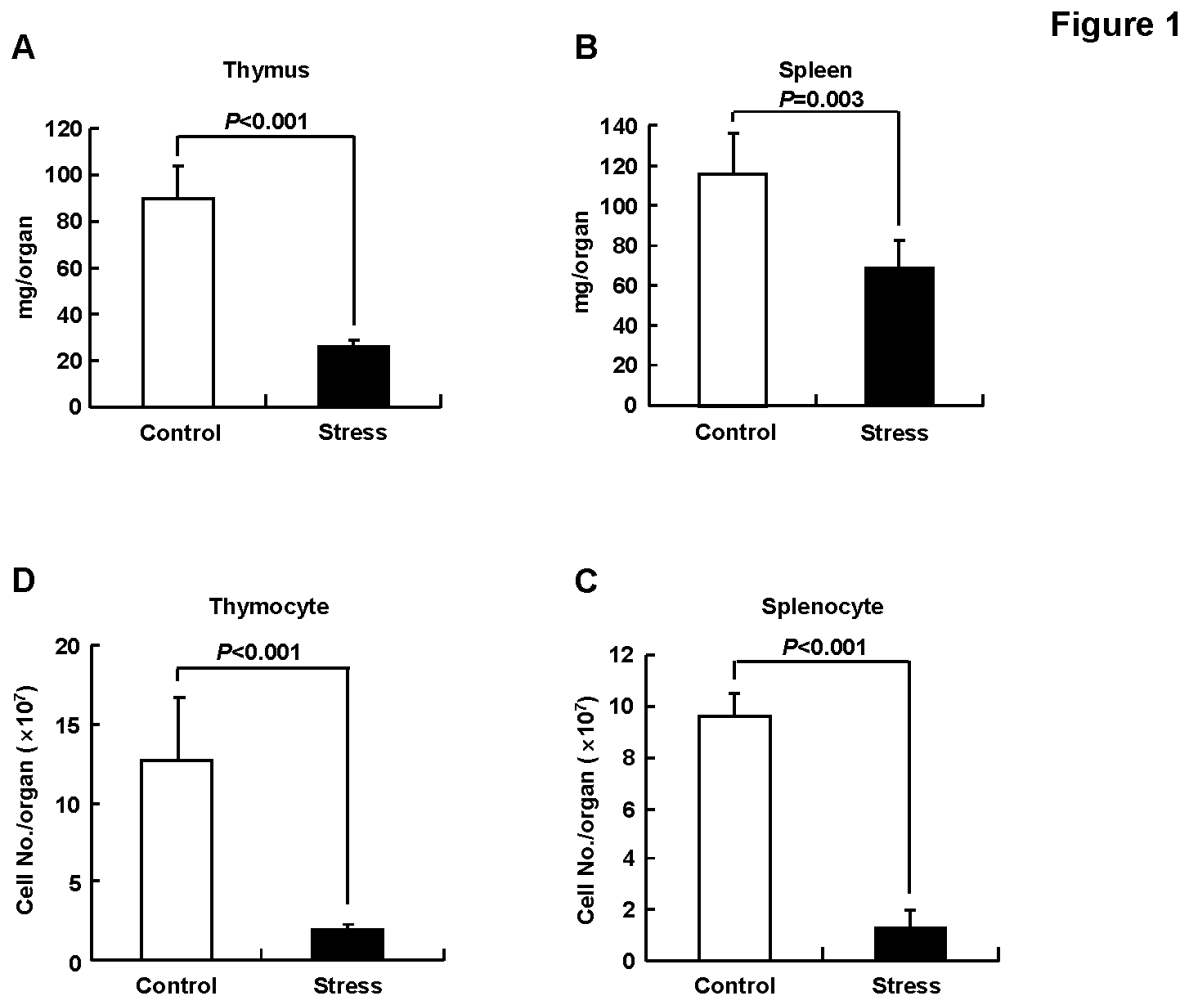
Repeated restraint sessions led to reduced organ weight and cell numbers. BALB/c mice were subjected to 5-days restraint stress or left untreated, n=5. Then the weight (A, B) and cell numbers of thymus and spleen (C, D) were measured. Results are expressed as mean ± SD.

### 8: Isolation of T cell subsets

To isolate CD4+ CD25- splenic cells, CD4+ T cells were first isolated (negative selection) from single-cell suspensions of spleens by using CD4+ T cell isolation kit II. Then the cells were incubated with CD25 microbeads. All CD25+ cells were depleted with a MS column. The purity of CD4+ CD25- T cells was > 95% as confirmed by flow cytometry.

**Figure 2:**
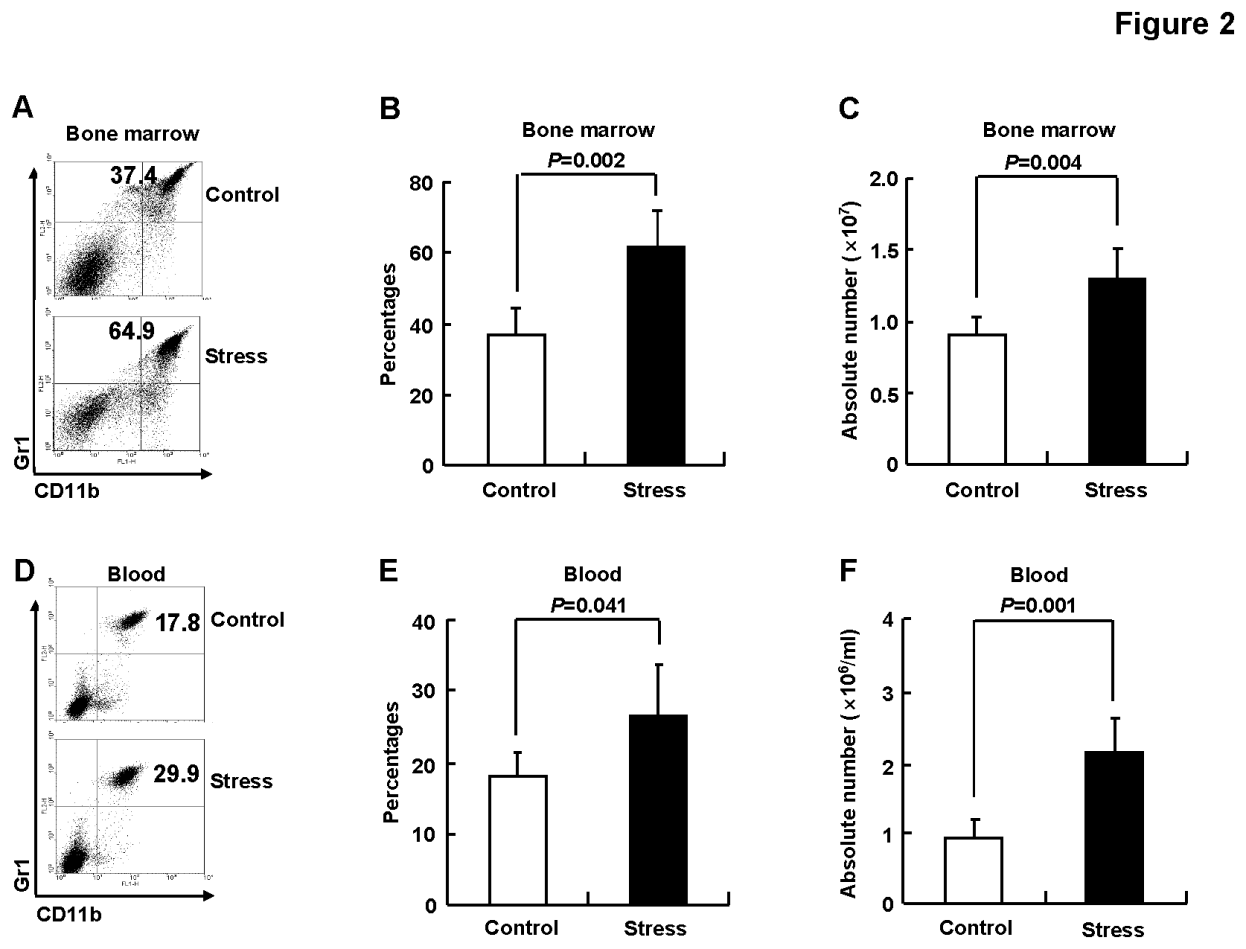
Chronic psychological stress induced the accumulation of CD11b+Gr1+ cells. BALB/c mice were subjected to 5-days restraint stress or left untreated, n=5. Bone marrow cells (A-C) and peripheral blood cells (D-F) were subjected to flow cytometry for CD11b and Gr1 staining. (A, D) Representative data. (B, E) Percentages. (C, F) Absolute numbers. Results are expressed as mean ± SD.

### 9: *In vitro* T cell suppression assays

CD4+ CD25- splenic cells were labeled by incubation at 10^6^/ml in RPMI 1640 medium with 0.1 µM CFSE at 37° for 20 min, washed and resuspended in RPMI 1640 medium containing 10% FBS. Purified bone marrow Gr1+ cells were then co-cultured with CFSE-labeled CD4+ CD25- splenic cells in a 96 well plate at the ratio of 1:1 or 0.5:1. In all the wells, the number of CD4+ CD25- splenic cells was kept constant (5×10^4^ cells/well). Stimulation was affected by antibodies against CD3 (precoated, 5 µg/ml) and CD28 (2 µg/ml) for 96 hours. Proliferation was assessed by flow-cytometric analysis of CFSE dilution.

### 10: ELISA

Serum samples were collected by bleeding after 5 days’ restraint stress. VEGF and PGE_2_ levels in the serum or cytokine levels in the supernatants were measured by using ELISA kits according to the manufacturer’s protocols.

### 11: Immunoblotting analysis

Whole cell lysates were prepared as previously described [27] and were resolved by SDS-PAGE before being transferred to nitrocellulose membranes. The membranes were then probed with various primary antibodies followed by peroxidase-conjugated secondary antibodies. Immunoreactive bands were visualized using an ECL chemiluminescence kit.

### 12: Real-time PCR

RNA was extracted by using TRIzol reagent. First-strand synthesis was performed with Oligo dT primers and reverse transcription was performed with M-MLV reverse transcriptase. Quantitative real-time PCR was performed using SYBR Green reagent in a real-time PCR machine Realplex 2 (Eppendorf, Hamburg, Germany). Reactions were performed in duplicate and GAPDH values were used to normalize gene expression. primers for murine arginase 1 were: 5′- ctccaagccaaagtccttagag-3′ (forward) and 5′-aggagctgtcattagggacatc-3′ (reverse), for murine iNOS were: 5′-gttctcagcccaacaatacaaga-3′ (forward) and 5′-gtggacgggtcgatgtcac-3′ (reverse), and for GAPDH were: 5′-ggcaaattcaacggcacagt-3′ (forward) and 5′-agatggtgatgggcttccc-3′ (reverse).

### 13: ROS production assays

The oxidation-sensitive dye 5-(and-6)-chloromethyl-2′,7′-dichlorodihydrofluorescein diacetate (carboxy-H2DCFDA) was used for the measurement of ROS production. Cells (10^6^/ml) were incubated in serum-free RPMI medium containing 2 µM carboxy-H2DCFDA, anti-CD11b-PE, and anti-Gr-1-PE-Cy5 at 37°C for 30 min. Cells were washed with PBS and were immediately subjected to flow cytometry to analyze the intensity of green fluorescence with 488nm excitation.

### 14: Statistical analysis

SPSS13.0 was used to analyze the data. The data were shown as mean ± standard deviations (SD). The student’s *t*-test was used to compare the difference between the two groups. Results were considered statistically significant at *P* < 0.05.

## Results and Discussion

### 1: Repeated restraint sessions led to reduced organ weight and cell numbers

Restraint stress paradigm provides a consistent physiological and psychological stress response. Mice subjected to ≥ four 12-h restraint sessions have been demonstrated to suffer from chronic psychological stress [2,3,7,9,11,14,15]. Chronic psychological stress is characteristic of a severe leukocytopenia and lymphocytopenia due to apoptotic loss of lymphocytes [14,16,28]. Consistent with previous reports, we found that mice subjected to five 16-h restraint sessions exhibited reduced mass of thymus and spleen (Figure 1A and B). Furthermore, the numbers of thymocytes and splenocytes significantly decreased in mice underwent five restraint sessions (Figure 1C and D). These data demonstrate that mice were indeed severely stressed after five 16-h restraint sessions.

### 2: Chronic psychological stress induced the accumulation of CD11b+Gr1+ cells

Repeated restraint sessions promote tumorigenesis and metastasis [2,3], delay wound healing [7], and compromise host defenses against various infections including *Listeria monocytogenes* [9], influenza virus [11], herpes simplex virus type 1 [14], and Theiler’s murine encephalomyelitis virus [15]. Accumulating evidence indicates that MDSCs are pluripotent, affecting both innate and adaptive immunity through direct contact and the secretion of various soluble factors [18,24]. Therefore, we next set out to explore whether the accumulation of MDSCs contributes to general immunosuppression associated with this model of chronic psychological stress. MDSCs are characterized by surface expression of both CD11b and Gr1. Because CD11b+Gr1+ cells are normally present in the bone marrow of healthy mice [29], the percentage and the absolute number of CD11b+Gr1+ cells in the bone marrow of control and stressed mice were analyzed. We found that chronic psychological stress induced a significantly higher percentage of CD11b+Gr1+ cells in the bone marrow (Figure 2A and B). Moreover, the absolute number of CD11b+Gr1+ cells in the bone marrow increased in mice underwent chronic psychological stress (Figure 2C). Similar accumulation of CD11b+Gr1+ cells was also seen in the peripheral blood of stressed mice (Figure 2D-F). However, the composition of lymphocytes in the spleen and peripheral blood mononuclear cells remained largely unchanged in stressed mice (data not shown). Together, our data indicate that chronic psychological stress induced the accumulation of CD11b+Gr1+ cells.

**Figure 3:**
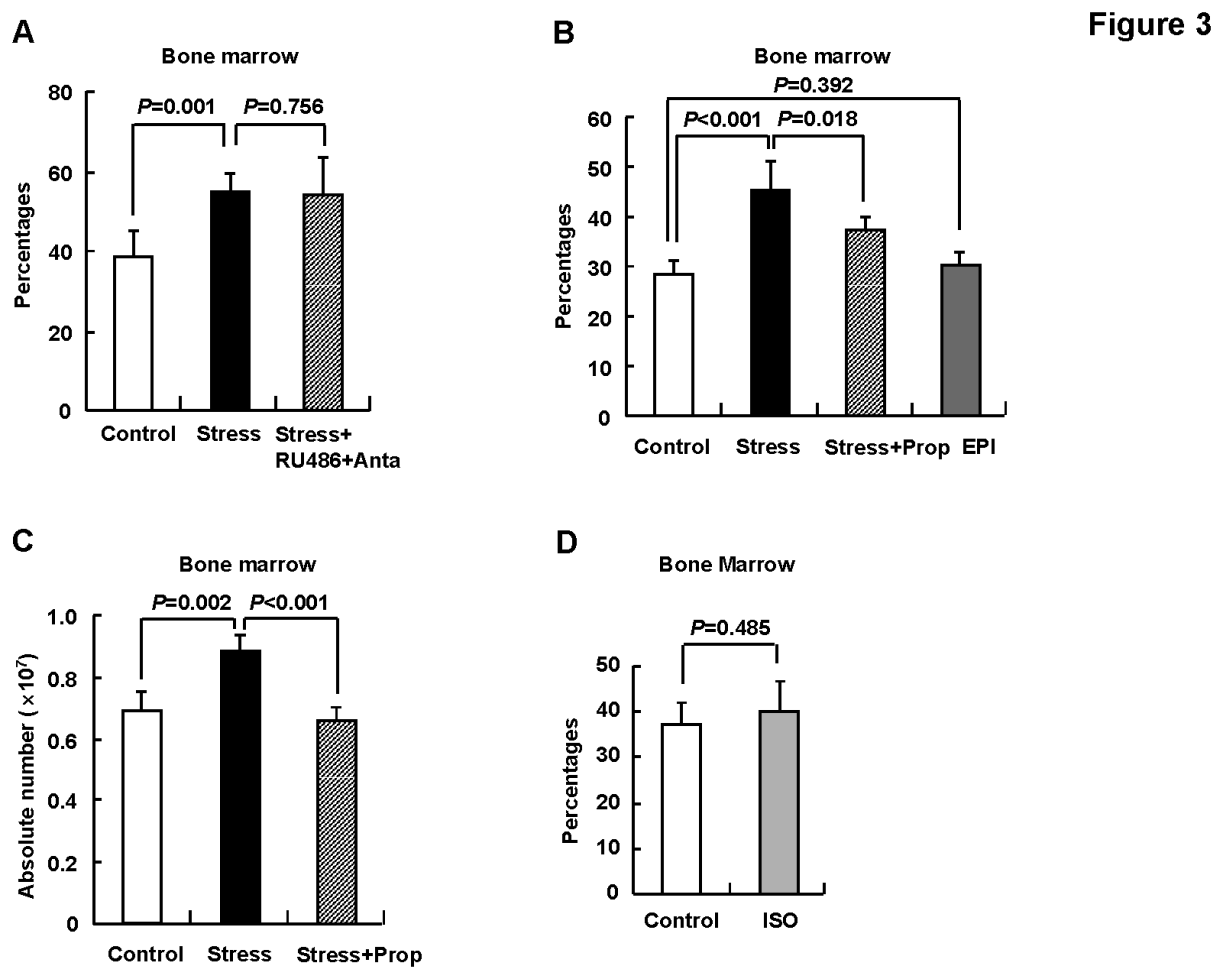
Catecholamines collaborated with other factors to induce the accumulation of CD11b+Gr1+ cells. BALB/c mice were subjected to 5-days restraint stress or left untreated, n=5. (A) One hour before each restraint session, mice were i.p injected with mifeprostone (RU486) and antalarmin (Anta), or same volume of solvent propylene glycol/ethanol. (B, C) One hour before each restraint session, mice were i.p injected with propranolol (Prop), epinephrine (EPI), or same volume of solvent PBS. (D) Mice were i.p injected with isoproterenol (ISO) or same volume of solvent PBS once daily for 5 days. Then, bone marrow cells were subjected to flow cytometry for CD11b and Gr1 staining. (A, B, D) Percentages. (C) Absolute number. Results are expressed as mean ± SD.

**Figure 4:**
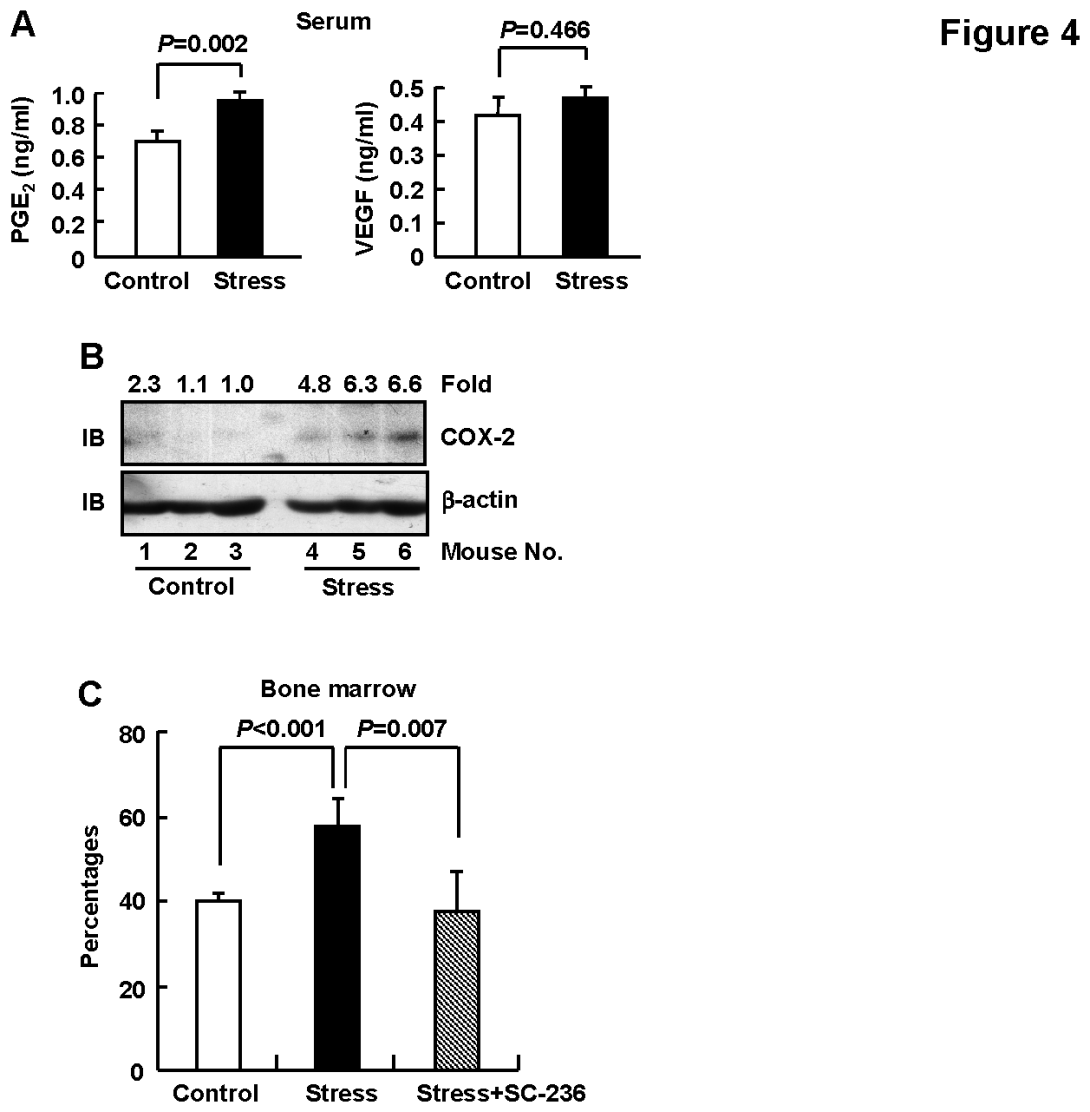
COX-2-PGE_2_ loop mediated the accumulation of CD11b+Gr1+ cells. BALB/c mice were subjected to 5-days restraint stress or left untreated. In some experiments, mice were i.p injected with SC-236 or same volume of solvent DMSO one hour before each restraint session. (A) Peripheral blood was sampled and serum PGE_2_ and VEGF levels were analyzed by ELISA. Results are expressed as mean ± SD, n=5. (B) Bone marrow Gr1+ cells were purified and subjected to immunoblotting, n=3. One representative of two independent experiments with similar results is shown. Densitometric readings are shown for COX-2 and normalized with β-actin (set the lowest ratio as 1.0). (C) Bone marrow cells were subjected to flow cytometry for CD11b and Gr1 staining. Percentages of CD11b+Gr1+ cells were shown. Results are expressed as mean ± SD, n=5.

### 3: Catecholamines collaborated with other factors to induce the accumulation of CD11b+Gr1+ cells

Chronic psychological stress is accompanied by continuously elevated glucocorticoids levels and exhaustive catecholamines release during each stress cycle [16]. Thus, it is of importance to explore whether glucocorticoids or catecholamines contribute to the accumulation of CD11b+Gr1+ cells under the condition of chronic psychological stress. We first assessed whether blockade of glucocorticoids peripheral action by systemic administration of the glucocorticoids receptor inhibitor mifeprostone (RU486) and antalarmin (a pharmacologic inhibitor of corticotropin-releasing factor). Unexpectedly, the coadministration of RU486 and antalarmin showed no effect on the increased percentage of bone marrow CD11b+Gr1+ cells under the condition of chronic psychological stress (Figure 3A). On the other side, β-adrenergic blockade with propranolol partially reversed the increased percentage of this subset (Figure 3B). The reversal of the accumulation of bone marrow CD11b+Gr1+ cells by propranolol was further confirmed by calculating the absolute number (Figure 3C). Then, we examined whether repeated infusion of epinephrine or isoproterenol, a nonspecific β-agonist [30], which mimics the persistent exposure to elevated levels of catecholamines, could also leads to the accumulation of CD11b+Gr1+ cells. However, daily injection with epinephrine or isoproterenol for five consecutive days had no effect on the percentage of CD11b+Gr1+ cells in the bone marrow (Figure 3B and D). Taken together, our data indicate that catecholamines collaborated with other factors to induce the accumulation of CD11b+Gr1+ cells.

### 4: COX-2-PGE_2_ loop mediated the accumulation of CD11b+Gr1+ cells

Previous studies have demonstrated that stress can lead to elevations in pro-inflammatory molecules some of which, such as PGE_2_ and VEGF, are associated with the development and/or promotion of immune suppressor cell populations [31-34]. Therefore, we firstly assessed the serum levels of PGE_2_ and VEGF in control and stressed mice. As displayed in Figure 4A, chronic psychological stress led to increased levels of circulating PGE_2_, but not VEGF. These data suggest that the accumulation of CD11b+Gr1+ cells under the condition of chronic psychological stress results from, at least partially, elevated levels of PGE_2_.

The biosynthetic pathway for PGE_2_ depends on the inducible COX-2 enzyme [35,36] and catecholamines contribute to COX-2 induction under the pro-inflammatory environment [37,38]. In this scenario, we checked the protein levels of COX-2 in the bone marrow CD11b+Gr1+ cells of control and stressed mice. Indeed, immunoblotting analysis revealed that chronic psychological stress led to increased levels of COX-2 protein (Figure 4B). As positive feedback between PGE_2_ and COX-2 plays a pivatol role in the induction and persistance of MDSCs [19], we next checked whether the disruption of COX-2-PGE_2_ feedback could reverse the accumulation of CD11b+Gr1+ cells under the condition of chronic psychological stress. As expected, COX-2 specific inhibitor SC-236 [39] reversed the increased percentage of this population (Figure 4C). Therefore, our data suggest that COX-2-PGE_2_ loop mediates the MDSCs accumulation downstream of catecholamines. COX-2-PGE_2_ loop might collaborate with other factors to induce MDSCs accumulation via promoting expansion, conversion, better persistence, and/or mobilization. Future studies are required to address this issue.

**Figure 5:**
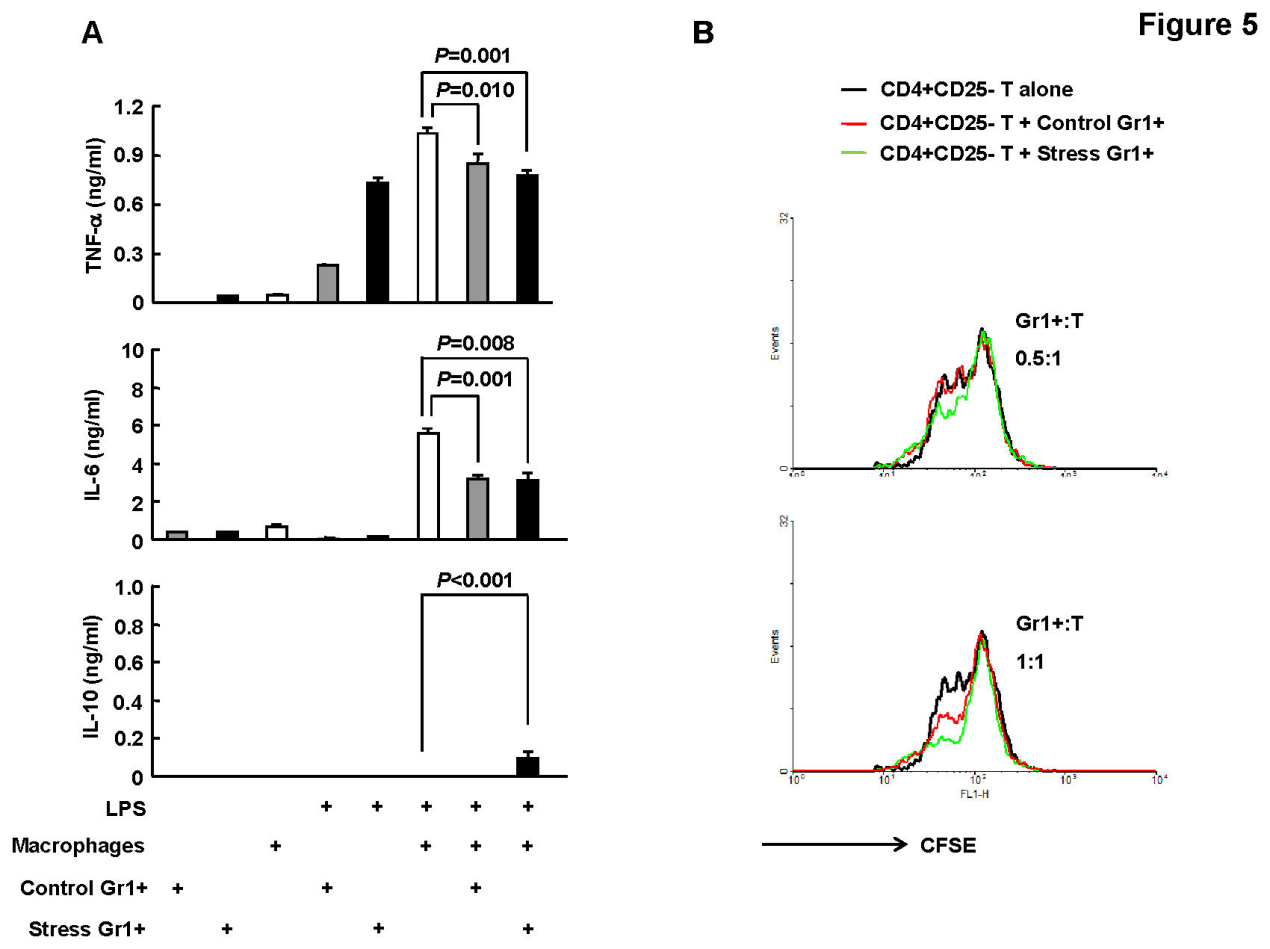
Accumulated CD11b+Gr1+ cells under the condition of chronic psychological stress were MDSCs. BALB/c mice were subjected to 5-days restraint stress or left untreated. Bone marrow Gr1+ cells were purified. (A) Bone marrow-derived macrophages were co-cultured with bone marrow CD11b+Gr1+ cells at a ratio of 1:4, and then were stimulated with or without 50 ng/ml LPS for 24 hours. Cytokine release was determined by ELISA. Results are expressed as mean ± SD, n=5. (B) CD4+ CD25- splenic T cells were purified and labeled with CFSE. Then, T cells were co-cultured with bone marrow CD11b+Gr1+ cells at a ratio of 1:1 or 0.5:1. Stimulation was affected by antibodies against CD3 and CD28 for 96 hours. Proliferation was assessed by flow-cytometric analysis of CFSE dilution. One representative of two independent experiments with similar results is shown.

**Figure 6:**
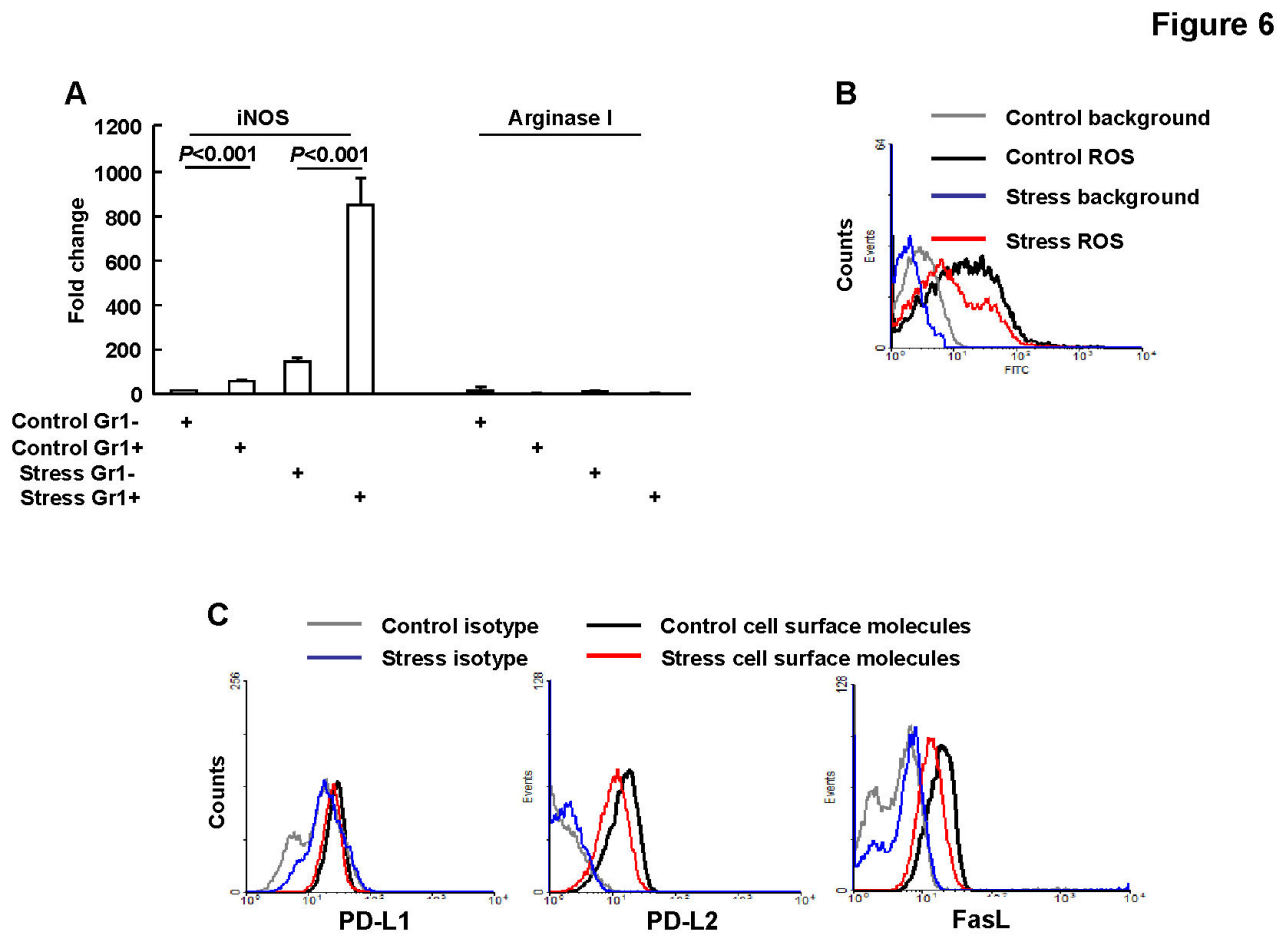
Accumulated MDSCs expressed multiple inhibitory molecules. BALB/c mice were subjected to 5-days restraint stress or left untreated, n=5. (A) Bone marrow Gr1+ cells were purified and subjected to real time PCR, Results are expressed as mean ± SD. (B) Bone marrow cells were subjected to flow cytometry for ROS production assays, representative data are shown. (C) Bone marrow cells were subjected to flow cytometry for PD-L1, PD-L2 and FasL expression, representative data are shown.

### 5: Accumulated CD11b+Gr1+ cells were MDSCs

Positive CD11b/Gr1 staining is not unique to MDSCs, especially in the bone marrow or the peripheral blood which has relatively high numbers of myeloid cells. MDSCs are best defined functionally, through their ability to suppress immune responses [29]. We found that bone marrow CD11b+Gr1+ cells isolated from both control and stressed mice showed significant expression of tumor necrosis factor-α (TNF-α), but marginal expression of interleukin-6 (IL-6) and IL-10 upon LPS stimulation (Figure 5A). Furthermore, this population isolated from either control or stressed mice similarly inhibited LPS-induced proinflammatory cytokine release such as TNF-α and IL-6 from primary macrophages (Figure 5A). Bone marrow CD11b+Gr1+ cells isolated from stressed mice produced more TNF-α upon LPS stimulation, but exhibited higher ability to induce the production of IL-10 in primary macrophages, as compared to their counterparts isolated from control mice (Figure 5A). Moreover, bone marrow CD11b+Gr1+ cells isolated from stressed mice directly suppressed the antigen-nonspecific (CD3/CD28) CD4+ T cell proliferative response more efficiently than their counterparts isolated from control mice (Figure 5B). Thus, bone marrow CD11b+Gr1+ cells isolated from either control or stressed mice were MDSCs and chronic psychological stress conferred a shift to their function.

### 6: Accumulated MDSCs expressed multiple inhibitory molecules

Multiple inhibitory molecules including PD-L1, PD-L2, FasL, IL-10, Arginase I, iNOS, and ROS have been attributed to the MDSCs-mediated suppression of immune responses [18,24]. Since bone marrow CD11b+Gr1+ cells isolated from stressed mice produced more TNF-α upon LPS challenge and IL-10 when co-cultured with microphages, we set out to analyze other mechanism(s) by which this population mediates suppression of immune responses. Real time PCR revealed that iNOS, but not Arginase I, was expressed by bone marrow CD11b+Gr1+ cells in control mice and chronic psychological stress led to upregulation of iNOS, but not Arginase I (Figure 6A). On the other hand, bone marrow CD11b+Gr1+ cells in either control or stressed mice showed similar ROS levels (Figure 6B) and similar PD-L2 and FasL expression, but exhibited no PD-L1 expression (Figure 6C). Thus, multiple mechanisms may contribute to the inhibitory roles of this population in either control or stressed mice. And these data again indicate that chronic psychological stress conferred a shift to the phenotype of MDSCs.

Previous studies suggest that CD11b+Gr1+ MDSCs in mice may accumulate in diverse pathological conditions including cancer, infectious diseases, sepsis, trauma, bone marrow transplantation, and some autoimmune diseases [40]. Recently, it has been revealed that patients with higher stress levels exhibited higher baseline numbers of MDSCs and reduced psychological stress in patients with stages II and III breast cancer leads to enhanced immune function, fewer recurrences and improved overall survival [25]. Data from our group further indicate a link between chronic psychological stress and MDSCs. Bone marrow CD11b+Gr1+ cells in control mice expressed iNOS/ROS/PD-L2/FasL, but not IL-10/Arginase I/PD-L1. Even though stress showed no effect on MDSCs subpopulations (data not shown), it did upregulate IL-10 and iNOS. The discrepancy between ROS and iNOS has been observed under certain circumstances [40]. Moreover, it is noteworthy that bone marrow CD11b+Gr1+ cells isolated from stressed mice produced more TNF-α upon LPS stimulation. Taken together, our data suggest that MDSCs contribute to chronic psychological stress-induced immunosuppression through an increase in the number and a shift to their phenotype and function.

## References

[1] Glaser R (2005) Stress-associated immune dysregulation and its importance for human health: a personal history of psychoneuroimmunology. Brain Behav Immun 19: 3–11. doi:10.1016/j.bbi.2005.10.012. PubMed: 15581732. 15581732 10.1016/j.bbi.2004.06.003

[2] Thaker PH, Han LY, Kamat AA, Arevalo JM, Takahashi R et al. (2006) Chronic stress promotes tumor growth and angiogenesis in a mouse model of ovarian carcinoma. Nat Med 12: 939–944. doi:10.1038/nm1447. PubMed: 16862152. 16862152 10.1038/nm1447

[3] Feng Z, Liu L, Zhang C, Zheng T, Wang J et al. (2012) Chronic restraint stress attenuates p53 function and promotes tumorigenesis. Proc Natl Acad Sci U S A 109: 7013–7018. doi:10.1073/pnas.1203930109. PubMed: 22509031. 22509031 10.1073/pnas.1203930109 PMC3345015

[4] Meddings JB, Swain MG (2000) Environmental stress-induced gastrointestinal permeability is mediated by endogenous glucocorticoids in the rat. Gastroenterology 119: 1019–1028. doi:10.1053/gast.2000.18152. PubMed: 11040188. 11040188 10.1053/gast.2000.18152

[5] Strike PC, Steptoe A (2004) Psychosocial factors in the development of coronary artery disease. Prog Cardiovasc Dis 46: 337-347. doi:10.1016/j.pcad.2003.09.001. PubMed: 14961456. 14961456 10.1016/j.pcad.2003.09.001

[6] Marucha PT, Kiecolt-Glaser JK, amd Favagehi M (1998) Mucosal wound healing is impaired by examination stress. Psychosom Med 60: 362-365 10.1097/00006842-199805000-00025 9625226

[7] Tymen SD, Rojas IG, Zhou X, Fang ZJ, Zhao Y et al. (2013) Restraint stress alters neutrophil and macrophage phenotypesduring wound healing. Brain Behav Immun 28: 207-217. doi:10.1016/j.bbi.2012.07.013. PubMed: 22884902. 22884902 10.1016/j.bbi.2012.07.013 PMC3878450

[8] Ben-Nathan D, Lustig S, Feuerstein G (1989) The influence of cold or isolation stress on neuroinvasiveness and virulence of an attenuated variant of West Nile virus. Arch Virol 109: 1-10. doi:10.1007/BF01310513. PubMed: 2558625. 2558625 10.1007/BF01310513

[9] Yamaoka Y, Kawakita T, Nomoto K (2000) Protective effect of a traditional Japanese medicine, Bu-zhong-yi-qi-tang (Japanese name: Hochu-ekki-to), on the restraint stress-induced susceptibility against Listeria monocytogenes. Immunopharmacology 48: 35-42. doi:10.1016/S0162-3109(00)00176-4. PubMed: 10822087. 10822087 10.1016/s0162-3109(00)00176-4

[10] Ortiz DC, Sheridan JF, Marucha PT (2003) Stress-induced changes in pathophysiology and interferon gene expression during primary HSV-1 infection. Brain Behav Immun 17: 329-338. doi:10.1016/S0889-1591(03)00027-8. PubMed: 12946655. 12946655 10.1016/s0889-1591(03)00027-8

[11] Hunzeker J, Padgett DA, Sheridan PA, Dhabhar FS, Sheridan JF (2004) Modulation of natural killer cell activity by restraint stress during an influenza A/PR8 infection in mice. Brain Behav Immun 18: 526-535. doi:10.1016/j.bbi.2003.12.010. PubMed: 15331123. 15331123 10.1016/j.bbi.2003.12.010

[12] Kopnisky KL, Stoff DM, Rausch DM. (2004) 2004) Workshop report: the effects of psychological variables on the progression of HIV-1 disease. Brain Behav Immun 18: 246-261. doi:10.1016/j.bbi.2003.08.003. PubMed: 15050652. 15050652 10.1016/j.bbi.2003.08.003

[13] Aberg KM, Radek KA, Choi EH, Kim DK, Demerjian M et al. (2007) Psychological stress downregulates epidermal antimicrobial peptide expression and increases severity of cutaneous infections in mice. J Clin Invest 117: 3339-3349. doi:10.1172/JCI31726. PubMed: 17975669. 17975669 10.1172/JCI31726 PMC2045593

[14] Freeman ML, Sheridan BS, Bonneau RH, Hendricks RL (2007) Psychological stress compromises CD8+ T cell control of latent herpes simplex virus type 1 infections. J Immunol 179: 322-328. PubMed: 17579052. 17579052 10.4049/jimmunol.179.1.322 PMC2367250

[15] Steelman AJ, Dean DD, Young CR, Smith R 3rd, Prentice TW et al. (2009) Restraint stress modulates virus specific adaptive immunity during acute Theiler’s virus infection. Brain Behav Immun 23: 830-843. doi:10.1016/j.bbi.2009.03.010. PubMed: 19348911. 19348911 10.1016/j.bbi.2009.03.010 PMC2710426

[16] Kiank C, Holtfreter B, Starke A, Mundt A, Wilke C et al. (2006) Stress susceptibility predicts the severity of immune depression and the failure to combat bacterial infections in chronically stressed mice. Brain Behav Immun 20: 359-368. doi:10.1016/j.bbi.2005.10.151. PubMed: 16330179. 16330179 10.1016/j.bbi.2005.10.151

[17] Cacioppo JT, Kiecolt-Glaser JK, Malarkey WB, Laskowski BF, Rozlog LA et al. (2002) Autonomic and glucocorticoid associations with the steady-state expression of latent Epstein-Barr virus. Horm Behav 42: 32-41. doi:10.1006/hbeh.2002.1801. PubMed: 12191645. 12191645 10.1006/hbeh.2002.1801

[18] Ioannou M, Alissafi T, Lazaridis I, Deraos G, Matsoukas J et al. (2012) Crucial role of granulocytic myeloid-derived suppressor cells in the regulation of central nervous system autoimmune disease. J Immunol 188: 1136-1146. doi:10.4049/jimmunol.1101816. PubMed: 22210912. 22210912 10.4049/jimmunol.1101816

[19] Obermajer N, Muthuswamy R, Lesnock J, Edwards RP, Kalinski P (2011) Positive feedback between PGE2 and COX2 redirects the differentiation of human dendritic cells towards stable myeloid-derived suppressor cells. Blood 118: 5498-5505. doi:10.1182/blood-2011-07-365825. PubMed: 21972293. 21972293 10.1182/blood-2011-07-365825 PMC3217352

[20] Fujita M, Kohanbash G, Fellows-Mayle W, Hamilton RL, Komohara Y et al. (2011) COX-2 blockade suppresses gliomagenesis by inhibiting myeloid-derived suppressor cells. Cancer Res 71: 2664-2674. doi:10.1158/0008-5472.CAN-10-3055. PubMed: 21324923. 21324923 10.1158/0008-5472.CAN-10-3055 PMC3075086

[21] Tartour E, Pere H, Maillere B, Terme M, Taieb J et al. (2011) Angiogenesis and immunity: a bidirectional link potentially relevant for the momitoring of antiangiogenic therapy and the development of novel therapeutic combination with immunotherapy. Caner Metastasis Rev 30: 83-95. doi:10.1007/s10555-011-9281-4. 10.1007/s10555-011-9281-4 21249423

[22] Van Ginderachter JA, Beschin A, De Baetselier P, Raes G (2010) Myeloid-derived suppressor cells in parasitic infections. Eur J Immunol 40: 2976-2985. doi:10.1002/eji.201040911. PubMed: 21061431. 21061431 10.1002/eji.201040911

[23] Nicholson LB, Raveney BJ, Munder M (2009) Monocyte dependent regulation of autoimmune inflammation. Curr Mol Med 9: 23-29. doi:10.2174/156652409787314499. PubMed: 19199939. 19199939 10.2174/156652409787314499

[24] Sander LE, Sackett SD, Dierssen U, Beraza N, Linke RP et al. (2010) Hepatic acute-phase proteins control innate immune responses during infection by promoting myeloid-derived suppressor cell function. J Exp Med 207: 1453-1464. doi:10.1084/jem.20091474. PubMed: 20530204. 20530204 10.1084/jem.20091474 PMC2901069

[25] Mundy-Bosse BL, Thornton LM, Yang HC, Andersen BL, Carson WE (2011) Psychological stress is associated with altered levels of myeloid-derived suppressor cells in breast cancer patients. Cell Immunol 270: 80-87. doi:10.1016/j.cellimm.2011.04.003. PubMed: 21600570. 21600570 10.1016/j.cellimm.2011.04.003 PMC3129455

[26] Lin H, Chen C, Chen BD (2001) Resistance of bone marrow-derived macrophages to apoptosis is associated with the expression of X-linked inhibitor of apoptosis protein in primary cultures of bone marrow cells. Biochem J 353: 299-306. doi:10.1042/0264-6021:3530299. PubMed: 11139394. 11139394 10.1042/0264-6021:3530299 PMC1221572

[27] Wang J, Tang R, Lv M, Zhang J, Shen B (2010) Selective unresponsiveness to the inhibition of p38 MAPK activation by cAMP helps L929 fibroblastoma cells escape TNF-α-induced cell death. Mol Cancer 9: 6. doi:10.1186/1476-4598-9-6. PubMed: 20070884. 20070884 10.1186/1476-4598-9-6 PMC2818697

[28] Depke M, Fusch G, Domanska G, Geffers R, Völker U et al. (2008) Hypermetabolic stress syndrome in repeatedly stressed BALB/c mice. Endocrinology 149: 2714-2723. doi:10.1210/en.2008-0038. PubMed: 18325986. 18325986 10.1210/en.2008-0038

[29] Sica A, Bronte V (2007) Altered macrophage differentiation and immune dysfunction in tumor development. J Clin Invest 117: 1155-1166. doi:10.1172/JCI31422. PubMed: 17476345. 17476345 10.1172/JCI31422 PMC1857267

[30] Zhang J, Bui TN, Xiang J, Lin A (2006) Cyclic AMP inhibits p38 activation via CREB-induced dynein light chain. Mol Cell Biol 26: 1223-1234. doi:10.1128/MCB.26.4.1223-1234.2006. PubMed: 16449637. 16449637 10.1128/MCB.26.4.1223-1234.2006 PMC1367190

[31] Song X, Krelin Y, Dvorkin T, Bjorkdahl O, Segal S et al. (2005) CD11b^+^/Gr-1^+^ immature myeloid cells mediate suppression of T cells in mice bearing tumors of IL-1β-secreting cells. J Immunol 175: 8200-8208. PubMed: 16339559. 16339559 10.4049/jimmunol.175.12.8200

[32] Bunt SK, Sinha P, Clements VK, Leips J, Ostrand-Rosenberg S (2006) Inflammation induces myeloid-derived suppressor cells that facilitate tumor progression. J Immunol 176: 284-290. PubMed: 16365420. 16365420 10.4049/jimmunol.176.1.284

[33] Huang Y, Chen X, Dikov MM, Novitskiy SV, Mosse CA et al. (2007) Distinct roles of VEGFR-1 and VEGFR-2 in the aberrant hematopoiesis associated with elevated levels of VEGF. Blood 110: 624-631. doi:10.1182/blood-2007-01-065714. PubMed: 17376891. 17376891 10.1182/blood-2007-01-065714 PMC1924481

[34] Tanaka K, Furuyashiki T, Kitaoka S, Senzai Y, Imoto Y et al. (2012) Prostaglandin E2-mediated attenuation of mesocortical dopaminergic pathway is critical for susceptibility to repeated social defeat stress in mice. J Neurosci 32: 4319-4329. 22442093 10.1523/JNEUROSCI.5952-11.2012 PMC3784244

[35] Huang M, Stolina M, Sharma S, Mao JT, Zhu L et al. (1998) Non-small cell lung cancer cyclooxygenase-2-dependent regulation of cytokine balance in lymphocytes and macrophages: Up-regulation of interleukin 10 and down-regulation of interleukin 12 production. Cancer Res 58: 1208-1216. PubMed: 9515807. 9515807

[36] Robertson RP (1998) Dominance of cyclooxygenase-2 in the regulation of pancreatic islet prostaglandin synthesis. Diabetes 47: 1379-1383. doi:10.2337/diabetes.47.9.1379. PubMed: 9726224. 9726224 10.2337/diabetes.47.9.1379

[37] Liu X, Wu WK, Yu L, Sung JJ, Srivastava G et al. (2008) Epinephrine stimulates esophageal squamous-cell carcinoma cell proliferation via beta-adrenoceptor-dependent transactivation of extracellular signal-regulated kinase/cyclooxygenase-2 pathway. J Cell Biochem 105: 53-60. doi:10.1002/jcb.21802. PubMed: 18452159. 18452159 10.1002/jcb.21802

[38] Schlachetzki JC, Fiebich BL, Haake E, de Oliveira AC, Candelario-Jalil E et al. (2010) Norepinephrine enhances the LPS-induced expression of COX-2 and secretion of PGE2 in primary rat microglia. J Neuroinflammation 7: 2. doi:10.1186/1742-2094-7-2. PubMed: 20064241. 20064241 10.1186/1742-2094-7-2 PMC2819253

[39] Jiang XH, Lam SK, Lin MC, Jiang SH, Kung HF et al. (2002) Novel target for induction of apoptosis by cyclo-oxygenase-2 inhibitor SC-236 through a protein kinase C-beta(1)-dependent pathway. Oncogene 21: 6113-6122. doi:10.1038/sj.onc.1205778. PubMed: 12203123. 12203123 10.1038/sj.onc.1205778

[40] Gabrilovich DI, Nagaraj S (2009) Myeloid-derived suppressor cells as regulators of the immune system. Nat Rev Immunol 9: 162-174. doi:10.1038/nri2506. PubMed: 19197294. 19197294 10.1038/nri2506 PMC2828349

